# An Optimized Method for Extraction and Characterization of Phenolic Compounds in *Dendranthema indicum* var. *aromaticum* Flower

**DOI:** 10.1038/s41598-019-44102-9

**Published:** 2019-05-23

**Authors:** Lijie Zhong, Zhiyang Yuan, Lin Rong, Yaohua Zhang, Guoxi Xiong, Yi Liu, Chao Li

**Affiliations:** 1 Technology Center of China Tobacco Hubei Industrial LLC, Wuhan, 430040 P.R. China; 2 Key Laboratory of Analytical Chemistry for Biology and Medicine (MOE), College of Chemistry and Molecular Sciences, Wuhan University, Wuhan, 430072 P.R. China; 3 National Key Laboratory of Crop Genetic Improvement, College of Plant Science and Technology, Huazhong Agricultural University, Wuhan, 430070 P.R. China; 4 Key Laboratory of Coal Conversion and Carbon Materials of Hubei Province, College of Chemistry and Chemical Engineering, Wuhan University of Science and Technology, Wuhan, 430081 P.R. China; 5 College of Chemistry and Material Sciences, Guangxi Teachers Education University, Nanning, 530001 P.R. China

**Keywords:** Chemical biology, Secondary metabolism, Mass spectrometry

## Abstract

*Dendranthema indicum* var. *aromaticum* plant has been widely used as herbal medicine in China, however, the material basis responsible for the therapeutic benefits remains largely unclear. This study aimed to provide an optimized method for extracting and characterizing phenolic compounds in *D*. *indicum* var. *aromaticum* flower. Firstly, an ultrasound-assisted method combined with central composite circumscribed (CCC) design was applied to optimize phenolic compound extraction. Ethanol-acetic acid (70%:2%, v/v) was selected as solvent, and the optimal extraction condition was: extraction temperature, 57 °C; solid/liquid ratio, 1:30 g/mL; extraction time, 20 min. Secondly, an effective and economic HPLC-PDA-ESI-MS^n^ method was established and validated for phenolic compound characterization and quantification. As a result, 14 phenolic compounds were identified, including 8 phenolic acids and 6 flavonoids, and for the first time, oleuropein derivatives, chrysoeriol, and tricin are reported in *D*. *indicum* var. *aromaticum* flower. The content of phenolics identified by HPLC-MS^n^ was 6.42 ± 0.32 mg/g DW. The optimized method for extraction and characterization of phenolic compounds has significant meaning to future pharmaceutical and medicinal research on *D*. *indicum* var. *aromaticum*, and the results in this study can provide references for herbal research.

## Introduction

*Dendranthema indicum* var. *aromaticum* is a new varietas of *Dendranthema*, growing in sunny area with an altitude of more than 2000 meters in Shen Nongjia area of Hubei province, China. The whole plant of the new varietas emits strong aroma, and the folk usually dry the leaves and petals to use as sachet. What’s more, *D*. *indicum* var. *aromaticum* plant is widely used as Chinese herbal medicine to prevent cold, treat headache, enteritis, constipation, coronary heart disease and hypertension.

Researchers reported that *D*. *indicum* var. *aromaticum* essential oil has strong anti-microbial and anti-oxidant activities^1^. *D*. *indicum* var. *aromaticum* is a good source of phenolics. Some flavonoids were isolated from this plant, including luteolin, apigenin, and acacetin^2^. Luteolin was reported to display excellent anti-oxidant, anti-inflammatory, and anti-allergic activities^3^. In various human cancer cell lines, cell cycle arrest and apoptosis were induced by adding acacetin into cell culture medium^4,5^. Many *in vivo* and *in vitro* investigations have revealed that intake of some phenolic compounds especially certain flavonoids contributes to the prevention of hypertension^6,7^. All these studies provide theoretical basis for the therapeutic benefits of *D*. *indicum* var. *aromaticum* plant, however, the material basis being responsible remains largely unclear. To date, research on *D*. *indicum* var. *aromaticum* mainly focused on chemical constituent of essential oil, but very little on its phenolics composition. To better explore values of *D*. *indicum* var. *aromaticum* in scientific research and in medicinal resource development, it makes sense to figure out the phenolic profile in *D*. *indicum* var. *aromaticum*.

As far as we know, currently there is no systematic method of extracting and analyzing phenolic compounds in *D*. *indicum* var. *aromaticum* flower. Therefore, the first objective of the present study was to optimize a high-efficiency method to extract phenolic compounds from *D*. *indicum* var. *aromaticum* flower. Ultrasound-assisted extraction, a commonly used technique of bioactive substances extraction from food products^8^ and plant materials^9–11^ was adopted in this study, in addition, response surface methodology was used to optimize the combination of various extraction conditions. To analyze phenolic compounds, a good HPLC analytical method must balance resolution, time cost and solvent cost. Therefore, the second objective of this study was to establish an effective and economic HPLC-PDA-ESI-MS^n^ analytical method to characterize phenolics composition in *D*. *indicum* var. *aromaticum* flower.

## Results and Discussion

### Optimization of phenolic compounds extraction

Extraction methods of phenolic compounds in plant material include soxhlet, microwave-assisted extraction, supercritical fluid extraction, ultrasound-assisted extraction, etc.^12–14^. With broad literature retrieval, ultrasound-assisted extraction was found to be a simple and effective method of phenoilcs extraction^8,10,15^, which does not require complicated equipment or technique and could greatly increase the extraction efficiency by strengthening the fragmentation process and assisting the release, diffusion, and dissolution of the components inside cells^11,16^. Furthermore, ultrasound-assisted extraction is more moderate and more secure to operate for possible future large-scale extraction. Therefore, ultrasound-assisted extraction was chosen in this study.

**Figure 1:**
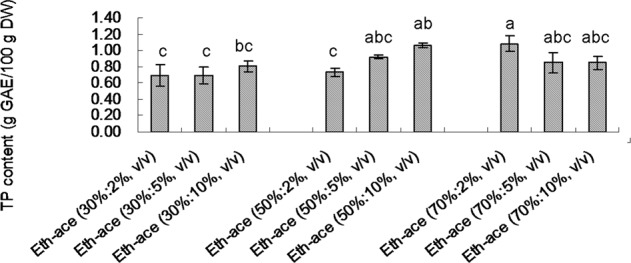
Effect of different proportion of ethanol and acetic acid combinations on TP content. Means of each two treatments were compared using least significant difference (LSD) statistic method. Lowercase letters a, b, and c were used to mark significance of difference (*p*  < 0.05). Same letters between treatments mean insignificant difference. Different letters between treatments mean significant difference.

Aqueous methanol, ethanol, and acetone were usually used for phenolic compounds extraction from botanical materials, especially from herbs^10,15^. Acid, for instance, hydrochloric acid, formic acid, and acetic acid was often used to acidify the extraction environment for better efficiency^15,17^. Considering the possible future industrial application of the phenolic extraction, relatively low toxic solvents ethanol and acetic acid were chosen in this study. Figure 1 shows the effect of different proportion of ethanol and acetic acid combinations on total phenolic (TP) content. In general, the extraction effect was increased by adding more acetic acid at lower concentration of ethanol (30% or 50%) but not at higher concentration of ethanol (70%). The highest TP content was obtained from ethanol-acetic acid (70%:2%, v/v) solvent, therefore, ethanol-acetic acid (70%:2%, v/v) was selected to be the extracting solvent in the following studies.

**Figure 2:**
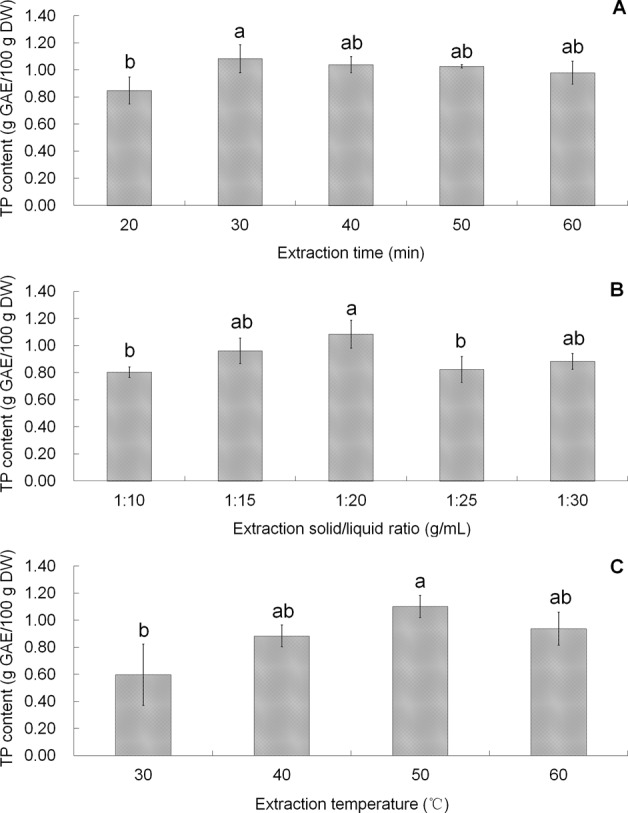
Effect of single factors (**A**) extraction time, (**B**) solid/liquid ratio, and (**C**) temperature on TP content. Means of each two treatments were compared using least significant difference (LSD) statistic method. Lowercase letters a and b were used to mark significance of difference (*p*  < 0.05). Same letters between treatments mean insignificant difference. Different letters between treatments mean significant difference.

To set up an appropriate CCC model, the ideal value range of independent variables should cover the inflection point of each independent variable. Therefore, the effect of each single factor on TP content was tested to obtain an approximate range for the CCC model construction, and the results were shown in Fig. 2. The increase of TP content was observed over the extraction time range (20∼30 min) (Fig. 2A), solid/liquid ratio range (1:10∼1:20 g/mL) (Fig. 2B), and extraction temperature range (30∼50 °C) (Fig. 2C). Moderate higher temperature and longer extraction time could enhance the solubility of phenolic compounds and accelerate the whole extraction process, however, degradation should always be considered when extraction time and temperature exceed an appropriate range. The inflection point of each variable was selected as central point of the CCC design: extraction time, 30 min; solid/liquid ratio, 1:20 g/mL; extraction temperature, 50 °C.

**Figure 3:**
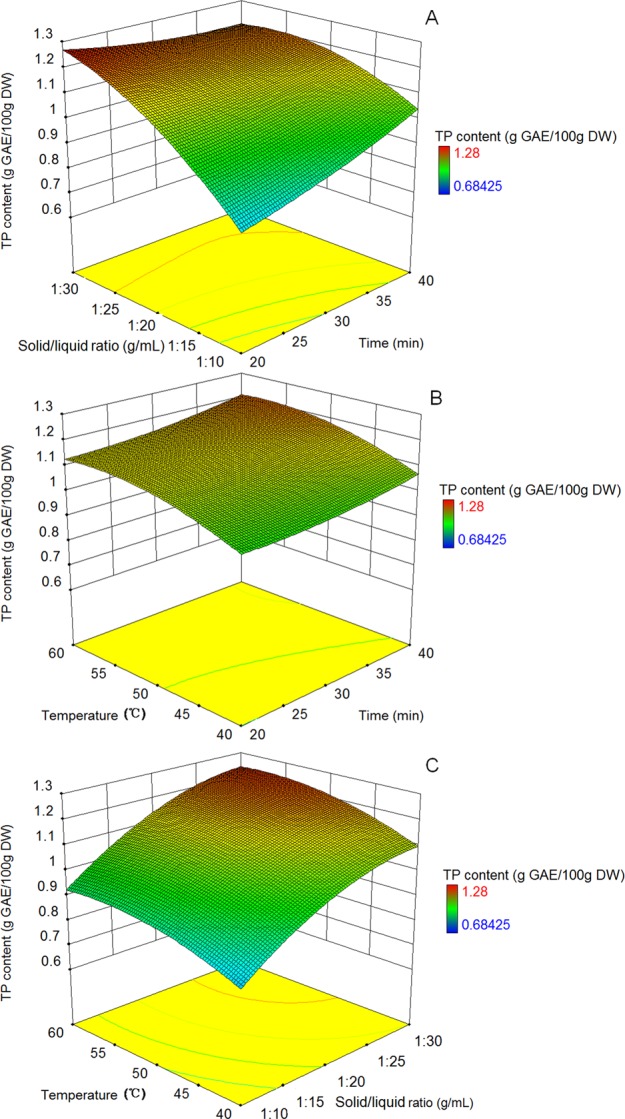
Interaction effect (**A**) between extraction time and solid/liquid ratio, (**B)** between extraction time and temperature, (**C)** between extraction solid/liquid ratio and temperature on TP content obtained from CCC design.

Extraction conditions and the corresponding results under CCC design were shown in Table 1. Statistical analysis revealed that quadratic model significantly fitted to the CCC design (*p* = 0.0002, R^2^ = 0.9211, statistic results were not shown). As seen in Fig. 3A, at certain extraction time, TP content rose with the increase of solid/liquid ratio. Further, relatively higher TP content was obtained when solid/liquid ratio tending to 1:30 with extraction time tending to 20 min simultaneously. As shown in Fig. 3B, TP content increased following temperature increasing at certain extraction time, and the rising trend was more obvious in 35 to 40 min extraction time range. In Fig. 3C, TP content went up following the rise of solid/liquid ratio under certain temperature. The more the solid/liquid ratio was close to 1:30 g/mL, the higher TP content was obtained in the temperature range of 50∼60 °C.

**Table 1:** Central composite circumscribed (CCC) design with five levels and three variables for phenolic compound extraction in *D*. *indicum* var. *aromaticum* flower and observed responses^a^.

Run order	Independent variables						TP content (g GAE^b^/100 g DW)
	Coded			Uncoded			
	Time (min)	Solid/liquid ratio (g/mL)	Temperature (°C)	Time (min)	Solid/liquid ratio (g/mL)	Temperature (°C)	
1	−1	−1	−1	20	1:10	40	0.71
2	1	−1	−1	40	1:10	40	0.93
3	−1	1	−1	20	1:30	40	1.16
4	1	1	−1	40	1:30	40	1.04
5	−1	−1	1	20	1:10	60	0.84
6	1	−1	1	40	1:10	60	1.03
7	−1	1	1	20	1:30	60	1.27
8	1	1	1	40	1:30	60	1.21
9	−1.68	0	0	13	1:20	50	1.11
10	1.68	0	0	47	1:20	50	1.28
11	0	−1.68	0	30	1:03	50	0.68
12	0	1.68	0	30	1:37	50	1.23
13	0	0	−1.68	30	1:20	33	0.89
14	0	0	1.68	30	1:20	67	1.13
15	0	0	0	30	1:20	50	1.02
16	0	0	0	30	1:20	50	1.20
17	0	0	0	30	1:20	50	1.07
18	0	0	0	30	1:20	50	1.26
19	0	0	0	30	1:20	50	1.15
20	0	0	0	30	1:20	50	1.07

^a^Experimental results for TP content are mean value of triplicates.

^b^Gallic acid equivalent.

The optimal ultrasound-assisted phenolic extraction condition obtained from the CCC model was shown in Table 2: extraction temperature, 57 °C; solid/liquid ratio, 1:30 g/mL; extraction time, 20 min. The model predicted a maximum response of 1.29 g GAE/100 g DW under optimal condition. TP content of 1.27 ± 0.08 g GAE/100 g DW obtained from real experiments validated the CCC model (Table 2). Mircea. *et al*. obtained 0.08∼0.15 g GAE/100 g DW phenolics from methanolic extract of *D*. *indicum* (L.) Des Moul (another variety of *D*. *indicum*) flower through ultrasound-assisted extraction^18^. The TP content we got in this study was about 10 times higher than that of *D*. *indicum* (L.) Des Moul, which reveals the potential of developing *D*. *indicum* var. *aromaticum* as a source of phenolics.

**Table 2:** Optimal condition and TP content obtained from prediction and real experiment under optimal condition.

Optimal condition			TP content (g GAE^a^/100 g DW)	
Time (min)	Solid/liquid ratio (g/mL)	Temperature (°C)	Predicted	Experimental^b^
20	1:30	57	1.29	1.27 ± 0.08

^a^Gallic acid equivalent.

^b^Mean ± SD (n = 3).

### Optimization of HPLC conditions

**Figure 4:**
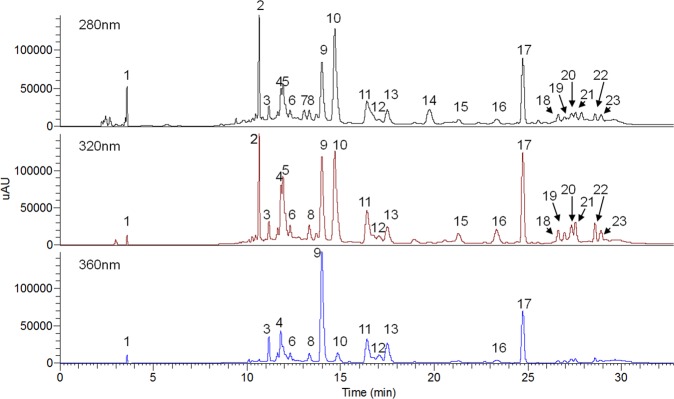
HPLC-PDA chromatograms of phenolic profile in *D*. *indicum* var. *aromaticum* flower under 280 nm, 320 nm, and 360 nm wavelength.

To analyze phenolic compounds by HPLC, acid was usually added in mobile phase^9,19^. Adding adequate amount of acid into mobile phase is beneficial to achieve complete separation, to lighten peak trailing, and to improve resolution of the compounds^20^. In this study, mobile phase B was fixed as acetonitrile, and mobile phase A was tested by various concentrations of formic acid (0.1%, 0.5%, and 1% v/v). It was found that using 0.1% or 0.5% formic acid did not result in the separation result as satisfactory as using 1% formic acid. Therefore, 1% formic acid was chosen in the following studies. Two 5 μm C18 columns (250 * 4.6 mm and 150 * 4.6 mm) were tested. By comparing with the longer column, using the shorter one could decrease flow rate from 1 mL/min to 0.6 mL/min and greatly shorten the elution time without affecting the separation result much. At last, various gradient methods were tried until a satisfactory chromatogram was obtained. Eventually, an effective and economic HPLC method was established. Figure 4 shows the HPLC-PDA chromatograms of phenolic compounds in *D*. *indicum* var. *aromaticum* flower.

### Identification of phenolic compounds

**Figure 5:**
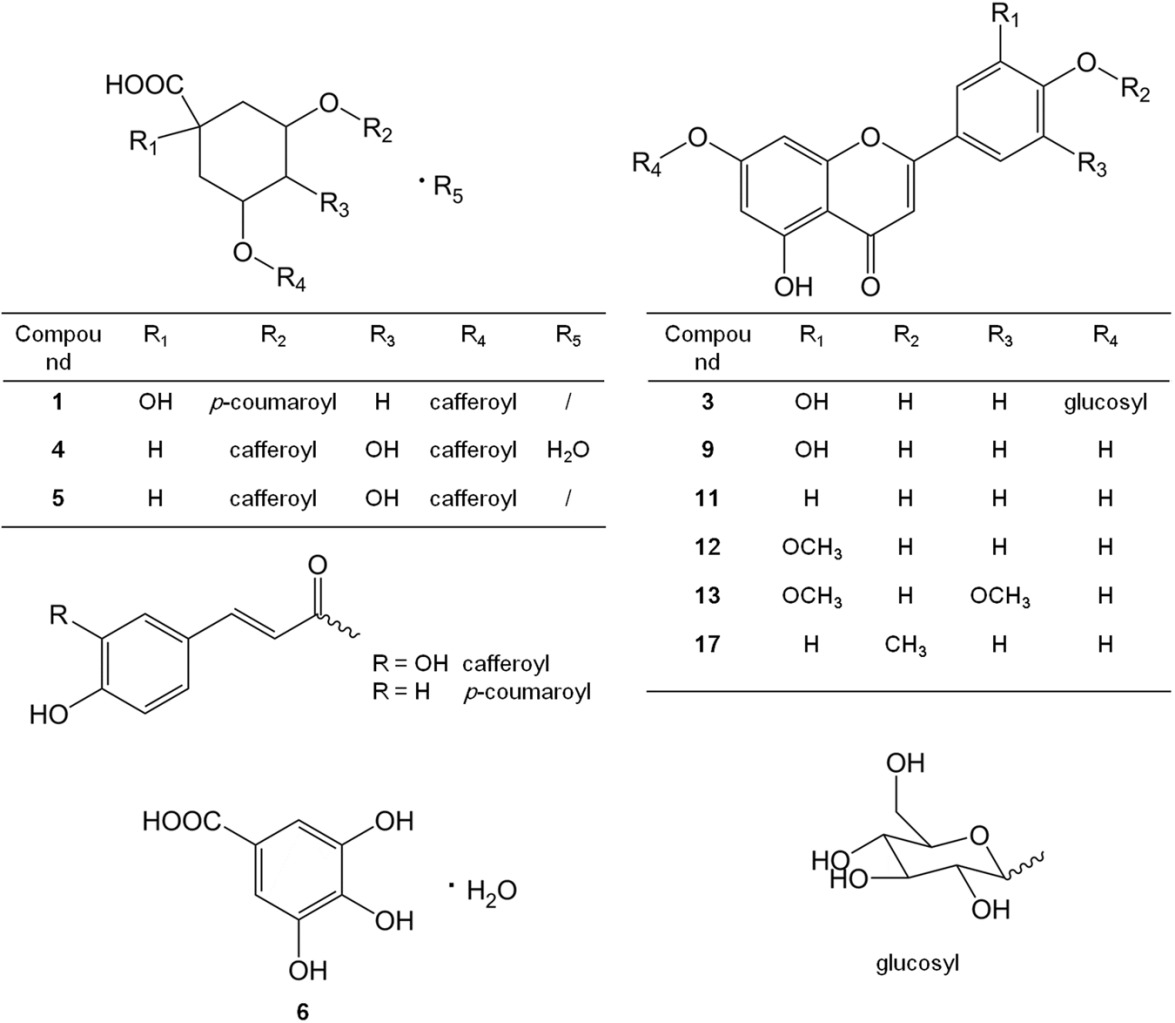
Definable chemical structures of identified phenolic compounds in *D*. *indicum* var. *aromaticum* flower.

In order to identify phenolic compounds, the external standard retention time and UV spectra from literatures were used to assess the target peaks preliminary, and the molecular structures were deduced based on MS and corresponding MS^n^ fragment signals. The identified phenolic compounds were classified into hydroxybenzonic acid group, hydroxycinnamic acid group, and flavonoid group in this study. Besides, several fatty acids were identified together with the phenolic compounds. A summary of the MS^n^ fragmentations of all compounds were shown (Table 3), and definable structures of identified phenolic compounds were drawn (Fig. 5).

**Table 3:** HPLC-MS^n^ information on phenolic compounds and hydroxy fatty acids identification in *D*. *indicum* var. *aromaticum* flower.

Peak No.	Rt (min)	UV λ_max_ (nm)	[M-H]−	MS^n^ *m/z* (% base peak)	Identification
1	3.59	260	499	MS^2^[499]: 481 (30), 353 (10), 191 (100), 173 (60)	3-*O*- Caffeoyl-5-*O-p*- coumaroylquinic acid
2	10.64	230, 300	685	MS^2^[685]: 539 (100), 523 (60), 665 (35); MS^3^[539]: 377 (100)	Coumaroyloleuropein
3	11.17	250, 340	447	MS^2^[447]: 285 (100)	Luteolin-*O*-glucoside
4	11.81	250, 330	533	MS^2^[533]: 353 (100), 335 (5), 191 (8), 173 (2); MS^3^[353]: 309 (1), 191 (100), 179 (16), 173 (6), 135 (4)	3,5-Dicaffeoylquinic acid monohydrate
5	11.92	240, 330	515	MS^2^[515]: 353 (100), 335 (5), 191 (4); MS^3^[353]: 191 (100), 179 (40), 173 (10), 135 (7)	3,5-Dicaffeoylquinic acid
6	12.30	250, 270, 320	187	MS^2^[187]: 169 (6), 125 (100)	Gallic acid monohydrate
7	13.05	250, 290	173	MS^2^[173]: 146 (8), 131 (100), 127 (15)	Shikimic acid isomer
8	13.32	250, 270, 320	569	MS^2^[569]: 551 (2), 525 (100), 459 (4), 417 (20), 391 (10); MS^3^[525]: 507 (16), 482 (18), 427 (10), 379 (28), 235 (15), 193 (100)	Methyoxyoleuropein isomer
9	13.99	250, 350	285	MS^2^[285]: 241 (35), 217 (20), 199 (25), 175 (25)	Luteolin
10	14.68	300	453	MS^2^[453]: 435 (2), 411 (6), 393 (100); MS^3^[393]: 363 (20), 249 (30), 231 (100), 205 (35); MS^4^[231]: 187 (100)	Prenyl-dimethoxy- caffeoyl-*p*-coumaric acid
11	16.41	270, 330	269	MS^2^[269]: 225 (40), 201 (12), 149 (10)	Apigenin
12	17.05	250, 330	299	MS^2[^299]: 284 (100); MS^3^[284]: 256 (100), 227 (10), 212 (5)	Chrysoeriol
13	17.49	250, 270, 340	329	MS^2^[329]: 314 (100); MS^3^[314]: 299 (100), 285 (10)	Tricin
14	19.74	260	659	MS^2^[659]: 615 (100), 591 (1), 573 (1), 505 (1), 265 (2); MS^3^[615]: 573 (100)	Unknown
15	21.30	240, 310	293	MS^2^[293]: 275 (100), 265 (25), 231 (45), 205 (40), 249 (90), 193 (28), 163 (20)	Monohydroxy- octadecaditrienoic acid
16	23.33	250, 310	/^a^	/^a^	Unknown
17	24.73	270, 330	283	MS^2^[283]: 269 (100)	Acacetin
18	26.61	270, 330, 350	/^a^	/^a^	Unknown
19	26.96	270, 310	313	MS^2^[313]: 295 (100), 277 (45), 215 (10), 183 (18)	Dihydroxy- octadecenoic acid
20	27.33	270, 320, 350	309	MS^2^[309]: 291 (50), 265 (10), 247 (100)	Dihydroxy- octadecatrienoic acid I
21	27.54	270, 320	309	MS^2^[309]: 291 (100), 265 (32), 247 (30)	Dihydroxy- octadecatrienoic acid II
22	28.58	310	295	MS^2^[295]: 277 (100), 265 (30)	Monohydroxy- octadecadienoic acid
23	28.91	270, 320, 350	297	MS^2^[297]: 279 (100), 251 (65), 223 (20)	Monohydroxy- octadecenoic acid

^a^No data available.

#### Hydroxycinnamic acid group

**Figure 6:**
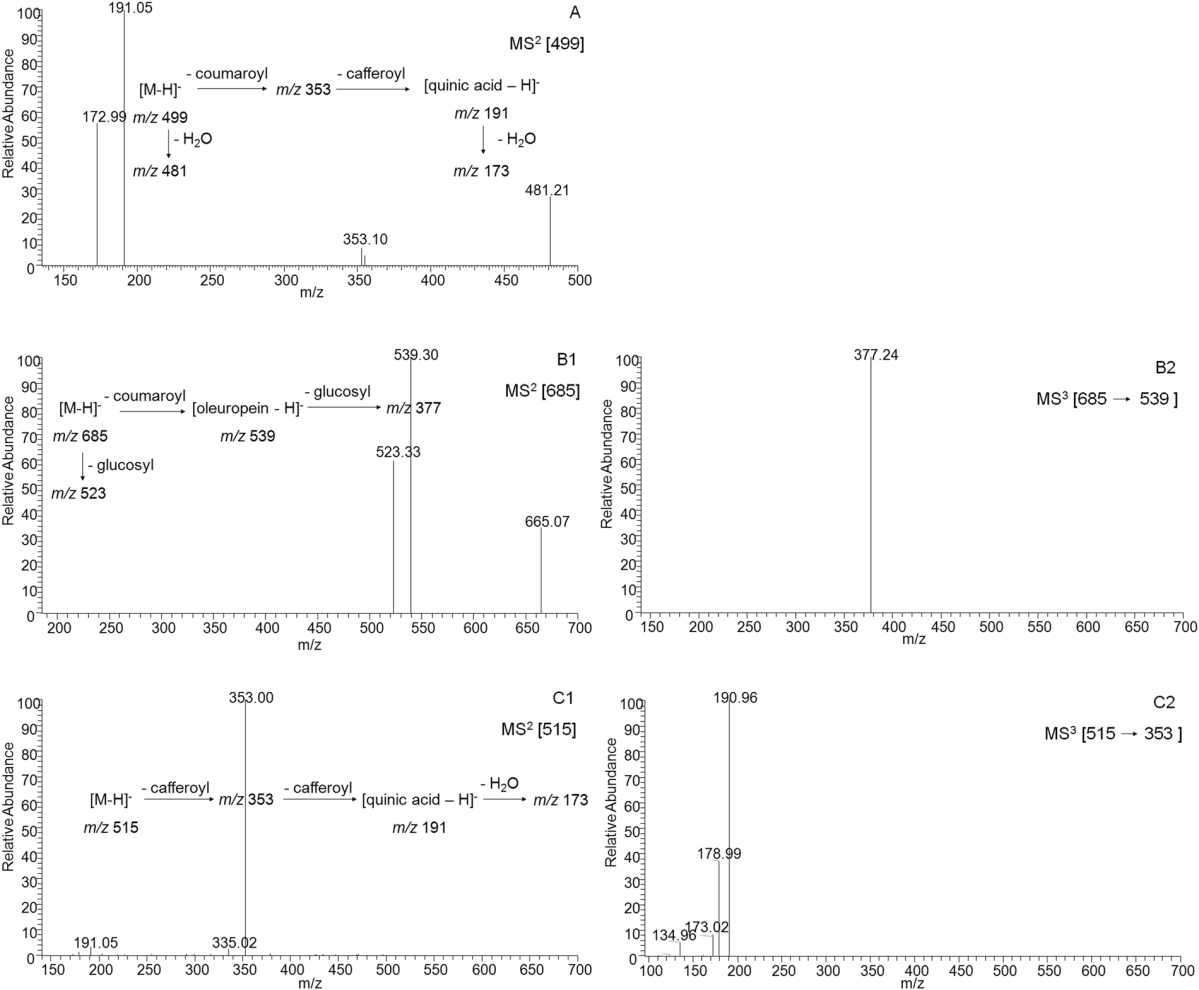
MS^n^ spectra and major fragmentations for the typical hydroxycinnamic acid derivatives in *D*. *indicum* var. *aromaticum* flower. (**A)** Compound **1** (3-*O*-caffeoyl-5-*O*-*p*- coumaroylquinic acid); (**B)** compound **2** (coumaroyloleuropein); (**C)** compound **5** (3,5-dicaffeoylquinic acid).

Compound **1** gave [M-H]^−^ ion at *m/z* 499, and the parent ion produced fragment ions at *m/z* 353 ([M-H-146]^−^) and 191([M-H-146-162]^−^) in MS2 spectrum, illustrating the losses of a coumaroyl moiety (146 Da) and a caffeoyl moiety (162 Da). In addition, fragment ion at *m/z* 191 revealed the existence of quinic acid moiety. Refering to previous report on ion fragments intensity characters of different esterification position of quinic acid structure^21^, compound **1** was identified as 3-*O*- caffeoyl-5-*O*-*p*- coumaroylquinic acid (Fig. 6A).

Compound **2** was identified as coumaroyloleuropein. As illustrated in Fig. 6B, firstly, the presence of its base ion at *m/z* 539 in MS2 with next base ion at *m/z* 377 in MS3 indicated the cleavage of a glucosyl from oleuropein^22,23^. Secondly, base ion at *m/z* 539 generated from parent ion at *m/z* 685 revealed the loss of a coumaroyl moiety, therefore, a structure of coumaroyloleuropein was deduced. Oleuropein is a bitter phenolic compound which mainly exists in green olives, olive leaves, and argan oil^24,25^. It is for the first time to report the presence of oleuropein derivative in *D*. *indicum* var. *aromaticum* flower.

For compound **8**, its parent ion at *m/z* 569 was 30 Da higher than that of oleuropein at *m/z* 539, which means that compound **8** might be methoxyoleuropein, but its MS^n^ spectrum did not give fragment ions as *m/z* 539 or 377 as expected according to literature reports^22,23^. Compound **8** was tentatively named as methoxyoleuropein isomer.

Spectrum of compound **5** showed parent ion at *m/z* 515 in MS and fragment ions at *m/z* 353 ([M-H-162]^−^) and 191([M-H-162-162]^−^) in MS2, indicating that two caffeoyl moieties (162 Da) cleaved from a quinic acid moiety. According to its characteristic ion fragment intensity pattern, compound **5** was deduced as 3,5-dicaffeoylquinic acid (Fig. 6C)^26^. Compound **4** gave almost the same ion fragment pattern as compound **5**, except that its parent ions at *m/z* 533 was 18 Da higher than that of compound **5** ([M-H]^−^ at *m/z* 515), therefore, it was deduced as 3,5-dicaffeoylquinic acid monohydrate.

Compound **10** was tentatively deduced to be prenyl-dimethoxy-caffeoyl-*p*-coumaric acid. The MS2 base fragment ion at *m/z* 393 [M-H-60]^−^ could be produced by the losses of two methoxy groups from [M-H]^−^ ion at *m/z* 453. Further, fragment ion at *m/z* 231 in MS3 would be produced by the cleavage of a caffeoyl moiety from the MS2 fragment ion at *m/z* 393, in addition, the fragment ion at *m/z* 231 in MS3 proved a prenylcoumaric acid moiety. Considering ion at *m/z* 393 in MS2, cleavage of two methyl groups from the preneyl moiety could produce the ion at *m/z* 363 in MS3.

#### Hydroxybenzoic acid group

Compound **6** exhibited a fragment peak [M-H-H_2_O]^−^ at *m/z* 169 with a base ion peak [M-H-H_2_O-CO_2_]^−^ at *m/z* 125, corresponding to MS signals produced by gallic acid standard. Furthermore, parent ion of compound **6** at *m/z* 187 (18 Da higher than that of gallic acid) indicated the peak was gallic acid monohydrate^19^.

Compound **7** had the same parent ion with shikimic acid at *m/z* 173, but its UV absorbance and MS2 fragments did not fit to previous report on shikimic acid^27^. The obtained information of MS fragments of compound **7** is insufficient to deduce its precise structure, so compound **7** was called shikimic acid isomer tentatively.

#### Flavonoid group

There were totally six flavonoids identified in *D*. *indicum* var. *aromaticum* flower in this study, and all of them are flavones and flavone derivatives.

Through comparison of retention time, UV spectrum, and MS^n^ patterns with external standards, luteolin, apigenin, and acacetin were identified for compounds **9**, **11**, and **17**, respectively. UV spectrum of compound **3** was pretty similar as luteolin, and the cleavage of a glucose from luteolin molecule could produce MS2 ion at *m/z* 285 ([M-H-162]^−^) from the parent ion at *m/z* 447. Thus, compound **3** was deduced as luteolin-*O*-glucoside.

The identical MS patterns of compound **12** ([M-H]^−^ 299; MS2[299]: 284 (100); MS3[284]: 256 (100)) and of compound **13** ([M-H]^−^ 329; MS2[329]: 314 (100); MS3[314]: 299 (100)) have been reported as chrysoeriol (3′-methoxy derivative of luteolin) and tricin in previous studies^19,28^. Chrysoeriol exists in many species of *Artemisia* genus^29^, and tricin occurs in rice bran and other grass specie such as wheat, maize, and barley^30^, both of these two flavones are for the first time to be reported in *D*. *indicum* var. *aromaticum* flower.

#### Other compounds

Several hydroxy fatty acids were identified together with phenolic compounds. Compounds **15**, **19**, **20**, **21**, **22**, and **23** were characterized to be hydroxy fatty acids, according to the phenomenon that water molecules cleaved from the aliphatic moiety consecutively in their MS2 spectra^31,32^. These peaks show up at end of the chromatogram, and most of them have longer retention time than the phenolic compounds except monohydroxy-octadecaditrienoic (compound **15**) which comes earlier than peak of acacetin (compound **17**) in the chromatogram (Fig. 4). Hydroxy fatty acids were found to have many biological activities, for instance, cytotoxicity and anti-inflammatory activity^33^, which might contribute to the therapeutic effects of *D*. *indicum* var. *aromaticum* plant, too.

### Method validation and phenolic compounds quantification

In this study, parameters of linearity, sensitivity, accuracy and precision were used for HPLC method validation. Results for method validation were summarized in Table 4. The correlation coefficients of the calibration curves were all over 0.99, which reflected that the detected concentration values were highly coincident with the real values. The method sensitivity was confirmed to be adequate as LOD and LOQ values were lower than 0.036 μg/mL and 0.109 μg/mL. Recovery rates of apigenin, acacetin, and luteolin obtained here varied from 99.50% to 102.19%, and the RSD values were lower than 1.84% and 2.70% for intra-day and inter-day experiments, respectively, indicating high accuracy and good precision of the analytical method in this study.

**Table 4:** Results for method validation.

No.	Standard	Linear regression curve	R^2^	LOD (μg/mL)	LOQ (μg/mL)	Recovery rate (%)^a^	RSD (%)^b^	
							Intra-day	Inter-day
1	Gallic acid	Y = 18817X − 10155	0.9996	0.014	0.042	/^c^	/^c^	/^c^
2	Caffeic acid	Y = 43686X − 19547	0.9998	0.010	0.030	/^c^	/^c^	/^c^
3	Apigenin	Y = 29655X + 6320.6	0.9999	0.013	0.040	99.50 ± 2.69	1.16	2.33
4	Acacetin	Y = 26463X + 9248.4	0.9999	0.013	0.041	102.19 ± 3.10	0.68	1.54
5	Luteolin	Y = 24041X + 44178	0.9989	0.036	0.109	100.47 ± 2.96	1.84	2.70

^a^Recovery rate (%) = [(measured amount after spiking − measured amount before spiking)/actual amount spiked] * 100.

^b^RSD (%) = (SD/Mean) * 100, where RSD means relative standard deviation and SD means standard deviation.

^c^Not analyzed.

As shown in Table 5, content of phenolics in *D*. *indicum* var. *aromaticum* flower identified by HPLC-MS^n^ was 6.42 ± 0.32 mg/g DW, consisting of 3.63 ± 0.17 mg/g DW of flavonoids and 2.79 ± 0.15 mg/g DW of phenolic acids. Among all the phenolic compounds in *D*. *indicum* var. *aromaticum* flower, luteolin (1.61 ± 0.11 mg/g DW) took up the highest proportion, around 25% of the TP content, which was found to be similar to the luteolin content (0.5∼2.1 mg/g DW) in flower of *D*. *indicum* (original variety of *D*. *indicum* var. *aromaticum*) reported in previous study^34^.

**Table 5:** Content of phenolic compounds in *D*. *indicum* var. *aromaticum* flower.

Peak No.	Compound	Group	Peak Area proportion (% total area)	Content^a^ (mg/g DW)
1	3-*O*-Caffeoyl-5-*O-p*- coumaroylquinic acid	Hydroxycinnamic acid	2.21	0.25 ± 0.01
2	Coumaroyloleuropein	Hydroxycinnamic acid	9.69	0.42 ± 0.03
3	Luteolin-*O*-glucoside	Flavonoid	2.49	0.16 ± 0.01
4	3,5-Dicaffeoylquinic acid monohydrate	Hydroxycinnamic acid	4.34	0.20 ± 0.00
5	3,5-Dicaffeoylquinic acid	Hydroxycinnamic acid	5.43	0.24 ± 0.00
6	Gallic acid monohydrate	Hydroxybenzoic acid	2.80	0.31 ± 0.02
7	Shikimic acid isomer	Hydroxybenzoic acid	3.07	0.34 ± 0.02
8	Methyoxyoleuropein isomer	Hydroxycinnamic acid	2.97	0.14 ± 0.01
9	Luteolin	Flavonoid	19.75	1.61 ± 0.11
10	Prenyl-dimethoxy-caffeoyl-*p*- coumaric acid	Hydroxycinnamic acid	20.88	0.89 ± 0.06
11	Apigenin	Flavonoid	9.12	0.57 ± 0.02
12	Chrysoeriol	Flavonoid	2.23	0.13 ± 0.01
13	Tricin	Flavonoid	3.95	0.24 ± 0.00
17	Acacetin	Flavonoid	11.09	0.92 ± 0.02
	Phenolic acids content			2.79 ± 0.15
	Flavonoids content			3.63 ± 0.17
	Content of phenolics identified by HPLC-MS^n^			6.42 ± 0.32

^a^Mean ± SD (n = 3).

Gong. *et al*. have isolated 20 mg acacetin from 1000 g dry flower of *D*. *indicum* var. *aromaticum* by absolute ethanol cold extraction and column chromatographyl^35^. Comparing with the acacetin content in this study (0.92 ± 0.02 mg/g DW, 0.92‰), the low yield of acacetin (0.02‰) in Gong’s work could be mainly caused by low extraction efficiency and high waste ratio during the isolation procedure. Previous studies stated that change of climate conditions (temperature, irradiation, rainfall, ect.) between years and different harvest periods could influence chemical composition and component content in plant material^36,37^. There were two phenolic compounds acacetin-7-O-β-D-glucopy ranoside and apignein-7-O-β-D-glucopy ranoside isolated from *D*. *indicum* var. *aromaticum* flower in Lu’s research by column chromatography and semi-preparative HPLC in 2009^2^ but not found in this study. Even though plant materials were harvested from the same location, certain degree of difference of chemical profile would exist between different material batches, which should be considered and accepted in research work.

## Conclusions

The phenolic compound composition of *D*. *indicum* var. *aromaticum* flower was studied extensively for the first time. Firstly, ethanol-acetic acid (70%:2%, v/v) was selected as extraction solvent. Ultrasound-assisted extraction method was optimized by CCC design, and under optimal condition (extraction temperature, 57 °C; solid/liquid ratio, 1:30 g/mL; extraction time, 20 min), 1.27 ± 0.08 g GAE/100 g DW TP content was obtained from *D*. *indicum* var. *aromaticum* flower. Secondly, an effective and economic HPLC-PDA-ESI-MS^n^ method was established, and the analytical method was validated by sensitivity, accuracy, and precision. As a result, 14 phenolic compounds were identified and quantified, including 8 phenolic acids and 6 flavonoids. For the first time, oleuropein derivatives, chrysoeriol, and tricin are reported in *D*. *indicum* var. *aromaticum* flower. In summary, the optimized method for extracting and characterizing phenolic compounds in *D*. *indicum* var. *aromaticum* flower has significant meaning for future pharmaceutical and medicinal research on *D*. *indicum* var. *aromaticum* plant, and the results in this work would provide references for future herbal research.

## Material and Methods

### Chemicals and materials

Eight standards were used in this work: gallic acid, caffeic acid, luteolin were purchased from Adamas Reagent (Shanghai, China); acacetin was purchased from TCI (Tokyo, Japan); apigenin was purchased from WAKO Pure Chemical Industries (Osaka, Japan); protocatechuic acid, ferulic acid, and hesperidin were purchased from Sigma-Aldrich (MO, USA). Acetonitrile and formic acid in HPLC grade were purchased from Fisher Chemical (Geel, Belgium). Ethanol and acetic acid in AR grade were purchased from Sinopharm Chemical Reagent Co., Ltd (Shanghai, China). Folin-Ciocalteu and Na_2_CO_3_ were purchased from Sigma-Aldrich (MO, USA).

Flower of *D*. *indicum* var. *aromaticum* was harvested in Shen Nongjia area of Hubei province, China. The plant species was identified by Prof. Keli Chen, Hubei University of Chinese Medicine, and authenticated by Herbarium, Kunming Institute of Botany, Chinese Academy of Sciences. The certificate of plant material authentication was provided as Supplementary. Plant material was harvested freshly, and spread at a shady, cool and well-ventilated place to dry naturally. Dry material was kept in −80 °C freezer before using.

### Sample extraction

Dry flowers were milled to a fine and uniform particle size by a YB-500A grander (Shanghai Lijian Machinery Co., Ltd, Shanghai, China). Half gram of the powder was mixed with certain volume of solvent, and followed with an ultrasound bath extraction (KQ-600DB, 40 kHz, Kunshan Ultrasonic Instruments Co., Ltd, China). The ultrasound working power was set at 360 W. External water circulated from a water bath to keep the extraction temperature stable. Centrifugation of 10,000 *g* was operated at 4 °C for 10 min after extraction, using an Allegra X-30R centrifuge (Bechman Coulter, Inc., Califonia, USA). Supernatant was used for TP content measurement or HPLC-MS analysis. All extraction experiments were operated in triplicate.

#### Selection of extraction solvent

Different concentrations of ethanol (30%, 50%, and 70%) and acetic acid (2%, 5%, and 10%) were mixed in orthogonal design. Half gram of weighed samples were ultrasound extracted with 10 mL various solvents at 40 °C for 30 min. Supernatant was collected after centrifugation as described above.

#### Selection of the range of extraction time

Half gram of weighed samples were ultrasound extracted with 10 mL ethanol-acetic acid (70%:2%, v/v) (i.e. V_ethanol_:V_acetic acid_:V_water_ = 70:2:28) solvent at 40 °C for different time (20, 30, 40, 50, and 60 min). Supernatant was collected after centrifugation as described above.

#### Selection of the range of solid/liquid ratio

Half gram of weighed samples were ultrasound extracted with ethanol-acetic acid (70%:2%, v/v) under different solid/liquid ratio (g/mL) (1:10, 1:15, 1:20, 1:25, and 1:30) at 40 °C for 30 min. Supernatant was collected after centrifugation as described above.

#### Selection of the range of extraction temperature

Half gram of weighed samples were ultrasound extracted with 10 mL ethanol-acetic acid (70%:2%, v/v) at different temperature (30, 40, 50, and 60 °C) for 30 min. Supernatant was collected after centrifugation as described above.

#### CCC design

Experiment was designed according to CCC model with five levels (−1.68, −1, 0, 1, and 1.68) and three variables (extraction temperature, solid/liquid ratio, and extraction time) to achieve the best variable combination for TP extraction. The CCC design consists of six central points, eight factorial points, and six axial points, generating 20 sets of experiments. Both coded and uncoded form of independent variables were shown in Table 1.

### Determination of TP content

Folin-Ciocalteu method reported in Cicco’s study^38^ was adopted to measure the TP content, with slight modifications. In brief, 150 μL of appropriately diluted extract, 150 μL of Folin-Ciocalteu (50%, v/v), and 1.2 mL of Na_2_CO_3_ (5%, m/v) were mixed. After two hours’ incubation at room temperature, absorbance under 760 nm was measured with a blank (150 μL of extraction solvent instead of the extract) using a nucleic acid/protein analyzer (Beckman Coulter, DU 730, CA, USA). Calibration curve was established using gallic acid standard. Results were recorded as gallic acid equivalent (g GAE/100 g DW). Absorbance experiments were operated in triplicate.

### HPLC-PDA and HPLC-ESI-MS^n^ conditions

Phenolic compounds profile in *D*. *indicum* var. *aromaticum* flower was seperated by HPLC-PDA in an Ultimate 3000 system (Thermo Fisher Scientific, Waltham, MA, USA). Phenolic compounds were firstly eluted by gradient program consisting of mobile phase A (0.1%, 0.5%, or 1% of formic acid) and mobile phase B (acetonitrile), and then 1% formic acid was chosen for further analysis. An Agilent C18 (5 μm, 250 * 4.6 mm) column and a Phenomenex Luna C18 (5 μm, 150 * 4.6 mm) column were tested secondly. The shorter column was chosen based on a comprehensive comparison of separation effect, elution time, and solvent cost. In addition, several gradient programs were tested until satisfactory separation results were achieved. Finally, a gradient elution program was modified by using mobile phase A (1% formic acid) and mobile phase B (acetonitrile) at 0.6 mL/min flow rate with 5 μL of injection volume. The gradient program was as follows: 5% B at 0–3 min; 5–40% B at 3–8 min; 40% B at 8–15 min; 40–50% B at 15–20 min; 50–95% B at 20–25 min; 95-5% B at 25–30 min; 5% B at 30–33 min. Column temperature was 30 °C. Wavelengths of 280 nm, 320 nm, and 360 nm were set for recording chromatograms, according to literature reports^15,39,40^.

HPLC-ESI-MS^n^ (Thermo Fisher Scientific, Waltham, MA, USA) was used to identify and characterize phenolic compounds. The same HPLC condition described above was used. Selected compounds were analyzed by MS2, MS3, till MS4 as needed. Negative mode of ionization was performed, and a mass range of *m/z* 50∼700 was covered for full scan. Collision gas was ultrahigh pure helium (He). The ionization parameters were set as follows:, 40 units/min of sheath gas (N_2_); 2 units/min of auxiliary gas (N_2_); 4.50 kV of spray voltage; 300 °C of capillary temperature; −1.00 V of capillary voltage; −8.77 V of tube lens offset voltage.

### Method validation and phenolic compounds quantification

Five calibration curves were established with different concentrations for different standards: 0.5, 5, 25, and 50 μg/mL for gallic acid (at 280 nm wavelength), caffeic acid (at 320 nm wavelength), and apigenin (at 320 nm wavelength); 5, 25, 50, and 100 μg/mL for acacetin (at 320 nm wavelength); 5, 25, 50, and 150 μg/mL for luteolin (at 360 nm wavelength). According to signal to noise ratios of 3 and 10, limit of detection (LOD) and limit of quantification (LOQ) were calculated, respectively, to check the method sensitivity.

To confirm method accuracy, apigenin, acacetin, and luteolin standards were used for recovery rate test. Three levels of each standard were spiked to 0.7 mL of sample extract: apigenin (10, 20, and 30 μg/mL), acacetin (15, 30, and 45 μg/mL), luteolin (25, 50, and 75 μg/mL).

To determine intra-day and inter-day precision, spiked samples with standards were analyzed at five different time points in one day and at the same time point for five consecutive days.

Since not all standards for each compound were commercially available, only apigenin, acacetin, and luteolin were quantified directly with their authentic standards, while other compounds were quantified by internal standards with similar structure and properties using relative response factor (RRF). The internal standard protocatechuic acid was used to quantify 3-*O*-caffeoyl-5-*O-p*-coumaroylquinic acid, gallic acid monohydrate, and shikimic acid isomer. The internal standard ferulic acid was used to quantify coumaroyloleuropein, 3,5-dicaffeoylquinic acid monohydrate, 3,5-dicaffeoylquinic acid, methyoxyoleuropein isomer, and prenyl-dimethoxy-caffeoyl-*p*-coumaric acid. The internal standard hesperidin was used to quantify tricin, luteolin-*O*-glucoside and chrysoeriol.

Fixed concentration (20 μg/mL) of internal standards were mixed with the sample extract and with the known compounds (25 μg/mL) mentioned above. Quantification of the unknown compounds was carried out according to the following formula:

$${\rm{Concentration}}\,{\rm{of}}\,{\rm{unknown}}\,{\rm{compound}}\,{\rm{in}}\,{\rm{solution}}({\rm{\mu }}g/\mathrm{mL})=(A/{A}_{i})\ast {C}_{i}/{\rm{RRF}}/R$$

where RRF = (*A*_*k*_/*A*_*i*_)/(*C*_*k*_/*C*_*i*_); *A*_*k*_ means peak area of known phenolic compound; *A*_*i*_ means peak area of internal standard; *C*_*k*_ means concentration of known phenolic compound; *C*_*i*_ means concentration of internal standard; *A* means peak area of unknown phenolic compound; *R* means recovery rate.

### Statistical analysis

For the single factor extraction tests, ANOVA was adopted and the TP content of different extraction treatments were analyzed under Tukey’s test by Minitab 17 (Minitab Inc., State College, PA, USA). Data were expressed as mean ± SD (*n* = 3). Statistic of the CCC design was performed using Design Expert 10 (Stat Ease Inc., Minneapolis, USA). ANOVA was adopted for analyzing model main effect and interaction between variables. *P* < 0.05 was considered as significant.

## Data Availability

The datasets generated and analyzed in the current study are included in the main text and the raw data are available from the corresponding author.
